# The risk of cardiovascular events following community-acquired sepsis: a nationwide cohort study in Sweden

**DOI:** 10.1017/S0950268826101174

**Published:** 2026-02-19

**Authors:** Hanna Wetterberg, Anton Nilsson, Adam Linder, Maria Lengquist, Attila Frigyesi, Jonas Sundén-Cullberg, Malin Inghammar

**Affiliations:** 1 Infection Medicine, Department of Clinical Sciences, Lund, Lund University, Lund, Sweden; 2 Epidemiology, Population Studies and Infrastructures (EPI@LUND), Department of Laboratory Medicine, Lund University, Lund, Sweden; 3 Centre for Economic Demography, Lund University, Lund, Sweden; 4 Department of Infectious Diseases, Skåne University Hospital, Lund, Sweden; 5 Anaesthesia and Intensive Care, Department of Clinical Sciences, Lund, Lund University, Lund, Sweden; 6 Intensive and Perioperative Care, Skåne University Hospital, Lund, Sweden; 7 Center for Infectious Medicine, Division of Infectious Diseases, Department of Medicine Huddinge, Karolinska Institutet, Stockholm, Sweden; 8 Department of Infectious Diseases, Karolinska University Hospital Huddinge, Stockholm, Sweden

**Keywords:** cardiovascular disease, critical care, epidemiology, long-term effects, mortality, readmission, sepsis

## Abstract

In this nationwide cohort study, we assessed the long-term risk of major cardiovascular events following intensive care unit (ICU) treatment for community-acquired sepsis and septic shock, compared to the general population. We included 20313 adults admitted to Swedish ICUs between 2008 and 2019, identified through national healthcare registries, and matched each case to 20 randomly selected population controls. Entropy balancing adjusted for baseline co-morbidities, healthcare utilization, and socio-demographics. The association between sepsis and subsequent cardiovascular events (hospitalizations or deaths due to myocardial infarction, heart failure, or cerebral infarction) was analysed using Cox proportional hazards models. Sepsis was associated with increased cardiovascular risk, particularly during the first year (days 0–30 adjusted hazard ratio [aHR] 6.1 (95% CI 4.7–7.9); days 31–90; aHR 2.4 (95% CI 1.8–3.2); days 91–365 aHR 1.4 (95% CI 1.2–1.6)), with risk persisting through years 2–5 (aHRs 1.1–1.3). Heart failure risk remained elevated across all intervals, while risks of myocardial and cerebral infarction were mainly short term. The highest relative risks were observed in patients without prior heart disease or with low baseline cardiovascular risk. These findings suggest that sepsis might be an independent and under-recognized driver of long-term cardiovascular disease, highlighting the need for preventive strategies.

## Introduction

Sepsis is a life-threatening organ dysfunction caused by a dysregulated host response to infection [1]. Although a leading cause of global mortality, the short-term mortality rate of sepsis has decreased in high-income countries in recent years [2, 3]. With an increasing number of patients recovering from sepsis, studying long-term clinical outcomes is becoming more crucial [4]. Observational studies with long-term follow-up have shown poor outcomes in individuals post-sepsis, including cognitive impairment, anxiety and depression, renal failure, and cardiovascular events, leading to higher rates of re-hospitalization and mortality [3]. Specifically, the association between sepsis and cardiovascular disease (CVD) is increasingly recognized [5–8].

In a previous study examining long-term mortality and morbidity following sepsis, our group found substantially increased long-term mortality and morbidity compared to matched population controls, with major causes of death and re-admission including infection, cancer, and cardiovascular disease [9]. However, that analysis grouped multiple cardiovascular conditions together, limiting insights into which specific diagnoses may primarily drive the elevated risk. Additional research is needed to clarify which types of cardiovascular diagnoses predominantly contribute to the increased risk, as few studies have stratified by underlying heart disease. Such stratification is important as results may otherwise obscure important variations in the risk of subsequent CVD across patient groups. Understanding which types of cardiovascular events sepsis patients are at risk for after the initial post-sepsis phase is needed to inform clinical guidelines and to develop targeted interventions for this growing population.

Despite increasing recognition of the long-term consequences of sepsis, structured approaches to post-sepsis care are often underdeveloped, meaning that healthcare providers have limited ability to offer interventions. Identifying high-risk patients and implementing preventive strategies is important for improving long-term quality of life and reducing subsequent complications, re-hospitalizations, and mortality.

This study aimed to investigate the risk of long-term cardiovascular mortality and morbidity in critically ill patients with sepsis in comparison to a matched community cohort.

## Methods

### Study design

Using data from the national quality register, the Swedish Intensive Care Registry (SIR) [10], we identified critically ill patients ≥18 years admitted to an intensive care unit (ICU) with a diagnosis of community-acquired sepsis between 2008 and 2019. This population was originally described in our previous study on long-term mortality and morbidity [9], and additional methodological details can be found therein. In the current analysis, we focused on cardiovascular outcomes by comparing sepsis patients with matched controls from the general population, using an entropy balancing design that accounted for co-morbidities, healthcare utilization, medication use, and socio-economic and demographic characteristics to ensure thorough adjustment for confounding factors.

The Ethical Review Board approved the study (Etikprövningsmyndigheten, Dnr. 2019-03284), which was reported according to the Strengthening the Reporting of Observational Studies in Epidemiology (STROBE) reporting guidelines [11].

### Study population

**Figure 1.**
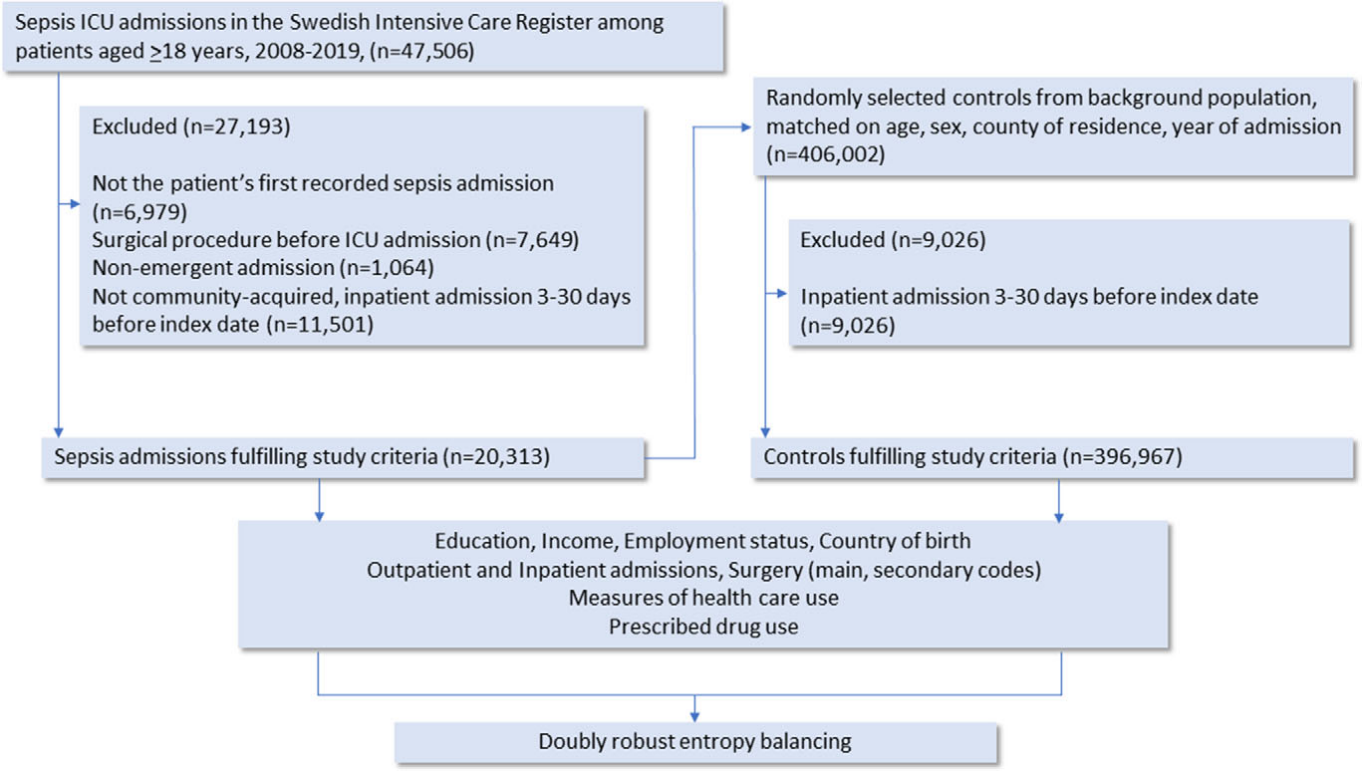
Flow chart of study patients collected from the Swedish Intensive Care Registry, 2008–2019, among patients aged ≥18 years, and the random selection of controls from the background population, matched for age, sex, county of residence, and year of admission. Values for exclusion criteria may not sum to the totals shown because some records were excluded for multiple reasons.

Community-acquired sepsis was defined as an ICU admission within 2 days of hospital arrival, with sepsis as the main or secondary diagnosis or an infection as the main diagnosis (Supplementary Table S1). The sepsis definition has been validated by medical records review [12]. For every sepsis case, Statistics Sweden matched 20 controls by birth year, sex, county of residence, and year drawn without replacement among individuals from the total population in Sweden, not observed as cases. Cases and controls were linked to administrative registers (Longitudinal integrated database for health insurance and labour market studies (LISA), Cause of Death [13], National Patient (NPR) [14], Prescribed Drug Registers [15]) for covariate and outcome data (Supplementary Table S2). Of 47506 patients with the targeted ICD codes identified in the Swedish Intensive Care Register, 20313 met the case definition of community-acquired sepsis after excluding patients with recent hospitalization (3–30 days before the index date), planned admission, surgeries, or previous sepsis episodes. To ensure comparability, controls were excluded if hospitalized 3–30 days before the index date, yielding 396976 controls (Figure 1).

### Outcome definition

The outcomes were incident myocardial infarction, heart failure, or cerebral infarction, defined as first hospitalization (primary NPR diagnosis) or underlying cause of death (Cause of Death Register) using International Statistical Classification of Diseases and Related Health Problem, 10th revision [ICD] codes I20–21, I23–25; I50.1, I50.9; and I.63. A composite outcome (any of the three) was also assessed.

### Statistical analysis

We estimated hazard ratios (HRs) for cardiovascular events using Cox regressions with robust standard errors, using time from ICU admission as the time scale and no minimum follow-up requirement. We censored participants at the date of the first CVD event, death, emigration, or end of follow-up (31 December 2019). Follow-up was divided into pre-specified intervals (0–30 days, 31–90 days, 91 days–1 year, 1–3 years, 3–5 years, and >5 years) to evaluate time-dependent risks. We applied doubly robust entropy balancing to control for confounding, yielding perfect covariate balance between cases and controls [16]. Our extensive balancing approach covered a comprehensive range of variables, including socio-demographic characteristics (age, sex, county of residence, country of birth, educational attainment, employment, and total disposable income per consumption unit), year of admission, medical history, measures of healthcare use, and prescription drug use. Missing values were addressed by creating a separate ‘missing’ category or setting them to zero for income. Weighting was performed at each landmark, based on baseline variables. From the 91-day landmark onwards, weights also incorporated certain post-sepsis conditions (Supplementary Table S2). This approach was employed to account for events occurring between landmarks while avoiding adjusting for outcomes caused by the sepsis event at baseline.

### Sub-group analyses

We conducted sub-group analyses by sex, age (</≥65 years), the Simplified Acute Physiology Score version 3 (SAPS3), a constructed disease risk score for cardiac events (Supplementary Method S1, Supplementary Table S3), infectious site (pneumonia, urinary tract, and other), and underlying heart disease (Supplementary Method S1, Supplementary Table S3). For each sub-group, we re-estimated weights to ensure balanced covariates within the sub-group.

To validate our case definition, we performed sensitivity analyses restricted to sepsis cases identified by infectious disease specialists from the National Quality Sepsis Registry (NQSR). Additionally, we explored alternative weighting methods to assess robustness and tested for effect modification by calendar year, by including interaction terms between sepsis status and year of admission within each landmark interval, evaluated using global Wald tests.

### Exploration of potential risk markers

To identify risk markers for CVD between days 31 and 365 after sepsis, we used Lasso Cox regression for variable selection and regularization [17]. The population was split into a training set for variable selection and a testing set for validation, considering demographics, co-morbidity, cardiovascular risk factors, and sepsis characteristics (Supplementary Method S2, Supplementary Table S4).

All analyses were conducted in Stata 18.0 (StataCorp) and the *ebalance* package [16].

## Results

Table 1 (previously published [9]) displays descriptive characteristics before re-weighting the controls. The median age was 70 (IQR 60–78), and 43% were female. Sepsis patients had lower socio-economic status, more co-morbidities, and higher healthcare use than unweighted controls. After reweighting, variables were balanced.

**Table 1.** Descriptive statistics of the sepsis patients and unweighted controls

Variable	Sepsis patients (*n* = 20313)	Controls, unweighted (*n* = 396976)
*Basic socio-demographics*		
Median and mean age (years)	70; 67.49 [60–78]	70; 67.30 [60–78]
Female sex (%)	43	43
*Cardiovascular and other major co-morbidities, past 5 years*		
Acute coronary syndrome (%)	5	2
Other ischemic heart disease (%)	0.2	0.1
Heart failure/cardiomyopathy (%)	15	4
Valve disorders (%)	5	2
Other heart disease, hypertonia, or cardiac surgery (%)	0.4	0.2
Vascular disease (%)	16	7
Cerebrovascular disease (%)	8	4
Thromboembolic disease (%)	3	1
Arrhythmia (%)	22	11
Pulmonary disease (%)	16	5
Diabetes (%)	21	8
Kidney disease (%)	10	2
Liver disease (%)	2	0.2
*Specific diagnoses, ever*		
Cardiac surgery (%)	12	8
Organ transplantation (%)	2	0.3
Childhood conditions (%)	2	0.4
*Specific pharmaceuticals, last year*		
Cardiac disease (%)	70	53
Lung disease (%)	18	10
Diabetes (%)	22	10
*Outcome information (general)*		
Median and mean follow-up time (years)	1.38; 2.62 [0.05–4.35]	4.22; 4.71 [1.98–7.07]
Died during follow-up (%)	56	15

*Note:* The table shows descriptive statistics before re-weighting the controls. Values within brackets are interquartile ranges. A more extensive version of this table can be found in our previous publication [9].

### Risk of cardiovascular events after sepsis

**Figure 2.**
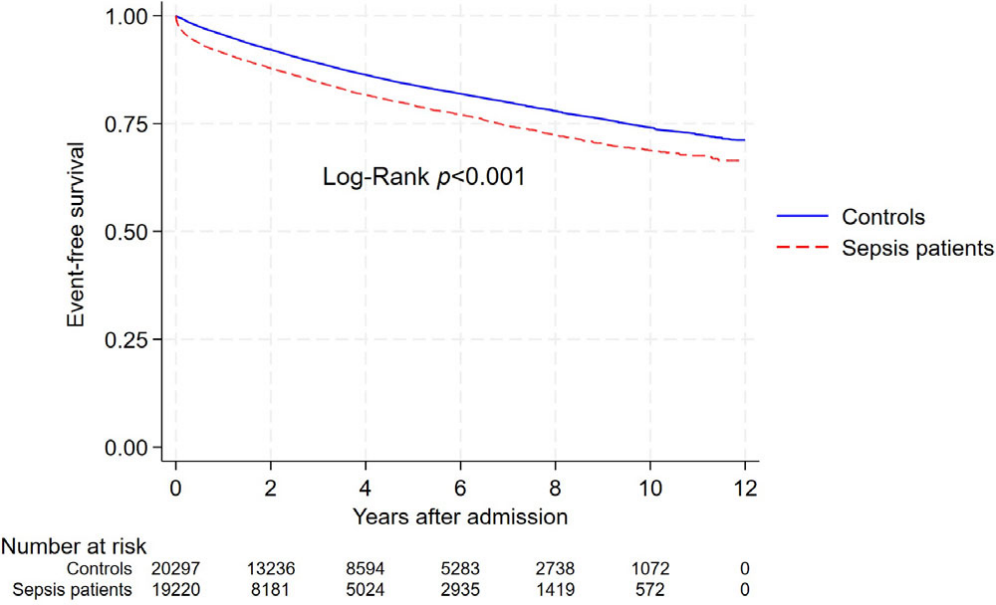
Weighted survival curve of major cardiovascular events (composite of hospitalization or death due to myocardial infarction, cerebral infarction, or heart failure) for sepsis patients and their matched controls. The log-rank test was used to compare survival distributions (*p* < 0.001). The numbers below the *x*-axis represent the number of individuals at risk in each group at various time points.

Over 12 years, the incidence rate (IR) of hospitalization or death due to major cardiovascular events was 51.0/1000 person-years (pyrs) among sepsis patients and 34.2/1000 pyrs in weighted controls (Figure 2, Supplementary Table S5). For sepsis patients surviving the first 30 days, the absolute cumulative risk of a cardiovascular event was 1.9% at 91 days, 5.9% at 1 year, 12.9% at 3 years, and 18.4% at 5 years, compared to 0.8%, 3.5%, 9.4%, and 14.0% in controls (Supplementary Table S6).

**Figure 3.**
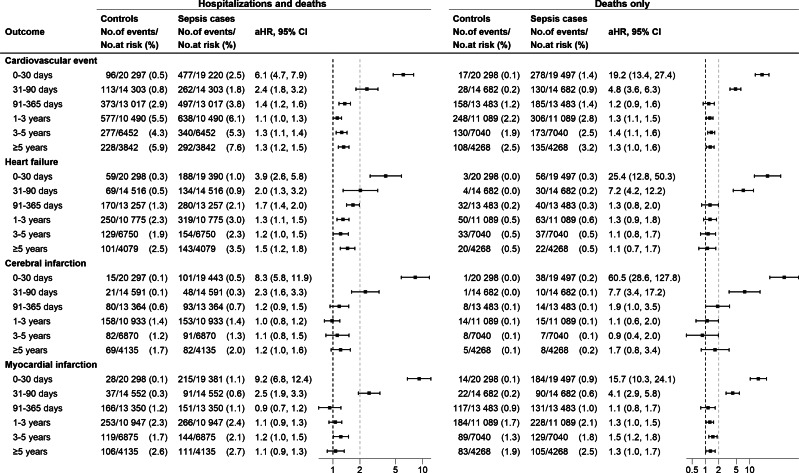
Adjusted hazard ratios for cardiovascular events, hospitalizations, and deaths in sepsis patients compared to matched controls across time intervals.

The adjusted hazard ratio (aHR) for any cardiovascular event was highest in the initial months after sepsis but remained elevated for at least 5 years, despite gradually decreasing over time (Figure 2). The aHR for heart failure remained elevated throughout the study period, particularly in the first year, while aHRs for myocardial and cerebral infarction were primarily elevated in the first 3 months, normalizing afterwards (Figure 3).

Deaths from cardiovascular events exhibited a similar pattern, with higher estimates during the first 3 months than for the combined outcome of hospitalization and deaths (Figure 3).

In sensitivity analyses, different weighting methods produced similar aHRs across most time points. Adding all post-sepsis co-morbidities produced slightly lower estimates for the time period of 5 years or more (Supplementary Figure S1). Tests for interaction with calendar year were not statistically significant across any Landmark period (Supplementary Table S7).

### Sub-group analyses

Sub-group analyses showed that aHRs were similar in males and females when comparing each group to its respective controls (Supplementary Figure S2). In younger patients, the aHRs were initially lower than in older, but became slightly higher from 31 days onwards, with overlapping intervals suggesting no statistically significant difference. Estimates for sepsis patients recorded in the NQSR and those not recorded were similar during the first 90 days, with overlapping confidence intervals. After this, the smaller recorded group showed no evidence of elevated risk, while those recorded had higher risks compared to controls. Among patients with and without heart disease, aHRs were higher in those with heart disease during the 0- to 30-day interval. However, at 31–90 days, aHRs were higher among those without, and the confidence intervals for this time did not overlap, suggesting a statistically significant difference in relative risk. After this, the aHRs began to converge with overlapping confidence intervals. For infectious sites, aHRs were elevated during the first 30 days across all groups. Between 31 and 90 days, the aHR remained elevated for lung and other infections, but declined for urinary tract infections. From 91 days onwards, the risks for all groups declined further, trending towards 1.0, with overlapping confidence intervals suggesting no clear differences between infectious sites. When stratified by SAPS3, the aHRs during the first year were higher for more severe sepsis but similar across levels afterward, though with overlapping confidence intervals. Finally, when stratified by cardiovascular risk score, aHRs showed a slightly higher relative risk among individuals with a low-risk score, but confidence intervals overlapped.

### Risk markers for cardiovascular event 31–365 days after sepsis

The Lasso regression models stratified patients into high- and low-risk groups for cardiovascular events, myocardial infarctions, and heart failure, showing statistically significant risk differences at 365 days and throughout follow-up (log-rank *p* < 0.001; Supplementary Figure S3). However, the model for cerebral infarction showed no predictive ability, with overlapping survival curves and no differences between risk groups (log-rank *p* = 0.641). Below, we highlight predictors with statistically significant associations; full details, including non-significant findings and confidence intervals, are available in the Supplementary Tables.

For cardiovascular events, older age was associated with an elevated risk (HR = 1.54, 95% CI: 1.22–1.94, per decade). Underlying co-morbidities such as heart failure (HR = 2.86, 95% CI: 1.78–4.59), arrhythmia (HR = 1.95, 95% CI: 1.27–2.99), and valve disorders (HR = 1.83, 95% CI: 1.11–3.02) also increased the risk. Elevated bilirubin within 24 h after ICU admission further raised the risk (HR = 2.90, 95% CI: 1.05–8.07), while absence of elevated creatinine was associated with lower risk (HR = 0.60, 95% CI: 0.38–0.95) (Supplementary Figure S4).

For myocardial infarction, hypoxemia at admission increased risk (HR = 2.70, 95% CI: 1.10–6.63). Each additional prescription with heart medication prior to admission was linked to higher risk (HR = 1.17 per medication, 95% CI: 1.01–1.35) (Supplementary Figure S5).

For heart failure, reliance on sick leave income was associated with an increase in risk (HR = 4.54, 95% CI: 1.11–18.64). Previous heart failure (HR = 5.11, 95% CI: 2.60–10.03), valve disorders (HR = 2.37, 95% CI: 1.26–4.46), and arrhythmia (HR = 2.13, 95% CI: 1.15–3.93) also emerged as predictors (Supplementary Figure S6).

## Discussion

In this population-based cohort study of critically ill patients with community-acquired sepsis in Sweden, we observed an elevated risk of major adverse cardiovascular events – including myocardial infarction, heart failure, and cerebral infarction – that persisted for at least 5 years. The highest hazard ratios were in patients without underlying heart disease, low baseline CVD risk, and those younger than 65, suggesting that sepsis is associated with increased cardiovascular risk, particularly for those without known cardiovascular co-morbidity.

Our findings align with previous studies showing increased cardiovascular risk following sepsis, with the highest risk during the first months and gradually declining [5, 7, 18–20]. The rigorous control for socio-demographic, clinical, and healthcare-related factors in our study strengthens the validity of these findings, minimizing potential confounding. While the elevated risk is consistently reported, estimates vary due to differences in methodologies, populations, and event definitions [5–7, 20]. Unlike many studies aggregating cardiovascular events, we identified a particularly elevated risk for heart failure, providing insights into processes linking sepsis to cardiovascular complications. The choice of comparison group also influences reported risk; studies using general population controls often yield higher risk estimates than studies using critically ill controls, which may underestimate the effects of sepsis and obscure the need for comprehensive follow-up and preventive measures for the individual [5, 7, 20].

We demonstrated an elevated risk of subsequent cardiovascular events in individuals without known underlying heart disease, where sepsis patients had almost a 10-fold higher risk than controls during days 31–90. A prior study also reported an increased risk in sepsis patients without pre-existing CVD, with younger individuals experiencing a particularly pronounced hazard ratio [20]. These findings indicate that sepsis acts as an independent risk factor for cardiovascular events, as younger patients have fewer underlying cardiovascular risk factors [20]. Additionally, a Swedish study found that elevated high-sensitive cardiac troponin T levels, a marker of myocardial injury, were independently associated with increased 1-year mortality in sepsis patients, suggesting sub-clinical myocardial injury during sepsis could contribute to adverse long-term outcomes. Together, these findings suggest early identification and monitoring of risk factors in sepsis patients are important, even in patients without known heart disease. However, the absolute risk for this group remained low, with 5.4% experiencing an adverse cardiovascular outcome during the first 5 years compared to 3.1% among controls.

Although a growing body of evidence suggests sepsis is an independent risk factor for cardiovascular events, disentangling the causal effects is difficult. The association between sepsis and CVD is complex, with CVD suggested to be both a risk factor and a consequence of sepsis [21] and one of the most common new diagnoses after sepsis [6]. Undiagnosed heart disease could predispose individuals to sepsis, while sepsis itself may accelerate the development or progression of cardiovascular disease, highlighting a potentially bi-directional relationship between sepsis and CVD.

Several potential overlapping mechanisms may underlie the observed association between sepsis and subsequent cardiovascular events, involving both longer term vascular and metabolic dysregulation and acute myocardial injury. Persistent low-grade inflammation may contribute to endothelial dysfunction and atherosclerosis, which could account for the longer term excess risk observed beyond the acute phase. Coagulation disturbances and myocardial depression may also contribute [7, 18], potentially explaining the pronounced early risk and the sustained elevation in heart failure observed in our study. Our exploratory analysis showed that, beyond baseline co-morbidity, markers of organ dysfunction within 24 h of admission, such as bilirubin and creatinine levels, may predict subsequent cardiovascular events, suggesting that acute systemic injury severity could identify patients at higher downstream cardiovascular risk. Similarly, a recent study showed that pneumonia, elevated troponin, and renal replacement therapy were associated with subsequent cardiovascular events [22].

From an intervention perspective, our results suggest that a targeted follow-up approach might be warranted, focusing on patients with a higher baseline risk profile and with markers of severe acute illness. Potential intervention pathways might include assessments of modifiable risk factors, such as blood pressure, lipids, and diabetes, as well as low-threshold access to evaluation of cardiac symptoms. In addition, exploratory analyses from a large observational study suggested that post-discharge statin use may be associated with a reduced risk of major cardiovascular events after sepsis [22]. Such observations highlight a potential pharmaceutical intervention strategy that warrants further study in future interventional or target trial-emulation studies.

The strengths of our study include a large nationwide sample of all ICU-treated sepsis patients in Sweden, long-term follow-up in registries with complete coverage, and robust adjustment for a comprehensive range of potential confounders through entropy balancing, allowing high precision and minimizing confounding bias. However, we could not exclude residual confounding from factors such as smoking, obesity, or physical activity, as these are unavailable in national databases. We only included sepsis patients treated in the ICU, so results are generalizable only to critically ill sepsis patients. ICU-treated sepsis represents the most severe end of the disease spectrum, and cardiovascular risk patterns may be weaker in less severe, non-ICU treated sepsis. However, given the substantially larger number of patients with sepsis managed outside the ICU, our findings may underestimate the overall population burden of post-sepsis cardiovascular disease. Misclassification of sepsis status is possible due to evolving definitions, with sepsis-3 introduced in SIR in 2018 [23]; however, a validation study showed 83% of the study population fulfilled the sepsis-3 criteria [12], and sensitivity analysis stratified by NQSR inclusion supported our sepsis definition’s reliability. In addition, the absence of interaction with calendar year across Landmark periods suggest that changes in diagnostic practice over time may not have influenced our risk estimates.

In conclusion, severe community-acquired sepsis was associated with an increased risk of hospitalization or death due to myocardial infarction, heart failure, or cerebral infarction for at least 5 years. Notably, the increased risk was observed even in individuals without underlying heart disease, in younger patients, and those with low baseline cardiovascular risk, indicating sepsis is associated with cardiovascular events independently of known underlying health factors. These findings underscore the need for preventive strategies against sepsis to reduce its long-term cardiovascular impact and the importance of implementing follow-up programmes that include cardiovascular monitoring. Such measures could reduce post-sepsis morbidity and mortality. Future studies should investigate if cardioprotective prophylaxis, guided by clinical factors such as high-sensitive cardiac troponin T levels, could help mitigate risk in targeted sepsis patients.

## Supporting information

10.1017/S0950268826101174.sm001 Wetterberg et al. supplementary material Wetterberg et al. supplementary material

## Data Availability

Data are available from the corresponding author upon reasonable request.

## Supplementary material

The supplementary material for this article can be found at http://doi.org/10.1017/S0950268826101174.
