# Supplementary material for: The risk of cardiovascular events following community-acquired sepsis: a nationwide cohort study in Sweden
Source: Epidemiol Infect. 2026 Feb 19;154:e32. doi: 10.1017/S0950268826101174 (PMC12976091; doi:10.1017/S0950268826101174)

The risk of cardiovascular events following community-acquired sepsis: A nationwide cohort study in Sweden

Supplementary material

Hanna Wetterberg, Anton Nilsson, Adam Linder, Maria Lengquist, Attila Frigyesi, Jonas Sundén-Cullberg, Malin Inghammar

Table of Contents

[Supplementary Methods 3](#_Toc219577533)

[Supplementary Table 1. Specific codes for infectious disease 3](#_Toc219577534)

[Supplementary Table 2. Variables included in the entropy balancing 4](#_Toc219577535)

[Supplementary Methods 1. Description of cardiovascular disease risk score and definition of underlying heart disease 8](#_Toc219577536)

[Cardiovascular disease risk score 8](#_Toc219577537)

[Underlying heart disease 8](#_Toc219577538)

[Supplementary Table 3. Variables included in the cardiovascular disease risk score and for definition of underlying heart disease 8](#_Toc219577539)

[Supplementary Methods 2. Explorative model for identifying risk markers of CVD in sepsis patients 9](#_Toc219577540)

[Supplementary Table 4. Variables included in the Lasso Cox Regression 9](#_Toc219577541)

[Supplementary Results 12](#_Toc219577542)

[Supplementary Table 5. Details of risk time and number of cardiovascular events and observations 12](#_Toc219577543)

[Supplementary Table 6. Absolute risks and risk differences for CVD 13](#_Toc219577544)

[Supplementary Figure 1. Sensitivity analysis: Comparison of hazard ratios for cardiovascular events using different weighting methods 15](#_Toc219577545)

[Supplementary Table 7. Test of interaction between sepsis status and calendar year 16](#_Toc219577546)

[Supplementary Figure 2. Weighted hazard ratios for subgroup analyses 17](#_Toc219577547)

[Supplementary Figure 3. The prognostic capacity of the models selected by Lasso regressions 19](#_Toc219577548)

[Risk markers by type of event, as selected by Lasso Cox regression 20](#_Toc219577549)

[Supplementary Figure 4. Risk markers for any cardiovascular event 31-365 days after admission 20](#_Toc219577550)

[Supplementary Figure 5. Risk markers for myocardial infarction 31-365 days after admission 21](#_Toc219577551)

[Supplementary Figure 6. Risk markers for heart failure 31-365 days after admission 22](#_Toc219577552)

# Supplementary Methods

## Supplementary Table 1. Specific codes for infectious disease

| **Disease** | **ICD 10-code** |
| --- | --- |
| Enteric infection | A00-A09 |
| Sepsis (bloodstream infections) | A40-A41, R57.2, R65.0-1 |
| Sexually transmitted infections | A50-A64, B20-B24 |
| Infections of the neurologic system, including the eye | A39, A80-89, B30, G00-02, G03.9, G40.0-2, G04.9, G05-08, G94.0, H00.0, H03, H04.3, H05.0, H06.1, H10.0, H10.2-3, H10.9, H13.0-1, H16.2, H16.8-9, H19.0-2, H22.0, H32.0, H44.0, H45.1 |
| Upper respiratory tract, including the ear | H60.0-3, H60.8-9, H61.0, H62, H66-67, H70, H73.0, H75.0, H83.0, H94.0, J00-06, J34.0, J36, J39.0-1 |
| Lower respiratory tract infections, including influenza | A15-19, A48.1, J09-22, J44.0-1, J47, J69.0, J85-86 |
| Infections of the heart and blood vessels | I30.1, I32.0-1, I33, I38-39, I40.0, I41.0-2, I43.0, I52.0-1, I68.1, I79.0-1, I98.0-1 |
| Infections of the digestive system including the liver (narrow definition) | B15-19, K04.4, K04.6-7, K10.2, K11.3, K12.2, K23.0-1, K61, K63.0, K65.0, K67, K75.0, K77.0, K83.0, K93.0, K93.1 |
| Infections of the genitourinary system | N08.0, N08.8, N10, N13.6, N15.1, N15.9, N16.0, N29.0-1, N30.0, N30.9, N33.0, N34.0-1, N37, N39.0, N41.0, N41.2-3, N43.1, N45.0, N45.9, N48.1-2, N49, N51, N61, N70-74, N75.1, N76.4, N77.0-1 |
| Infections of the skin and soft tissue | A46, L00-03, L05.0, L08, L30.3 |
| Infections of bone, joints and connective tissue | M00-01, M46.2-5, M49.0-2, M60.0, M63.0-2, M65.0, M68.0, M71.0-1, M72.5-6, M73.0-1, M86, M90.0-2 |
| Infectious complications | T79.3, T80.2, T81.4, T82.6-7, T83.5-6, T84.5-7, T85.7, T87.4, T88.0 |
| Other infections | A20-28, A30-38, A42-49, A65-69, A70-79, A92-99, B00-09, B25-27, B33-99, D70.9, D73.3, E06.0, E32.1 |
| Note. Classification according to: Gustav T *et al.* Hospitalisations with infectious disease diagnoses in somatic healthcare between 1998 and 2019: A nationwide, register-based study in Swedish adults. *Lancet Reg Health Eur*. 2022;16:100343 | |

## Supplementary Table 2. Variables included in the entropy balancing

| **Medical history < 5 years of index**  **Main and additional discharge diagnoses**  **Inpatient and Outpatient Registry** | | **ICD-10-SE^1^, ATC^2^, or procedure^3^ code (ICD-10 codes unless otherwise specified)** |
| --- | --- | --- |
| Enteric infection | | A00-A09 |
| Sepsis (bloodstream infections) | | A40-A41, R57.2, R65.0-1 |
| Sexually transmitted infections | | A50-A64, B20-B24 |
| Infections of the neurological system, including the eye | | A39, A80-89, B30, G00-02, G03.9, G40.0-2, G04.9, G05-08, G94.0, H00.0, H03, H04.3, H05.0, H06.1, H10.0, H10.2-3, H10.9, H13.0-1, H16.2, H16.8-9, H19.0-2, H22.0, H32.0, H44.0, H45.1 |
| Upper respiratory tract, including the ear | | H60.0-3, H60.8-9, H61.0, H62, H66-67, H70, H73.0, H75.0, H83.0, H94.0, J00-06, J34.0, J36, J39.0-1 |
| Lower respiratory tract infections, including influenza | | A15-19, A48.1, J09-22, J44.0-1, J47, J69.0, J85-86 |
| Infections of the heart and blood vessels | | I30.1, I32.0-1, I33, I38-39, I40.0, I41.0-2, I43.0, I52.0-1, I68.1, I79.0-1, I98.0-1 |
| Infections of the digestive system including the liver (narrow definition) | | B15-19, K04.4, K04.6-7, K10.2, K11.3, K12.2, K23.0-1, K61, K63.0, K65.0, K67, K75.0, K77.0, K83.0, K93.0, K93.1 |
| Infections of the genitourinary system | | N08.0, N08.8, N10, N13.6, N15.1, N15.9, N16.0, N29.0-1, N30.0, N30.9, N33.0, N34.0-1, N37, N39.0, N41.0, N41.2-3, N43.1, N45.0, N45.9, N48.1-2, N49, N51, N61, N70-74, N75.1, N76.4, N77.0-1 |
| Infections of the skin and soft tissue | | A46, L00-03, L05.0, L08, L30.3 |
| Infections of bone, joints and connective tissue | | M00-01, M46.2-5, M49.0-2, M60.0, M63.0-2, M65.0, M68.0, M71.0-1, M72.5-6, M73.0-1, M86, M90.0-2 |
| Other infections | | A20-28, A30-38, A42-49, A65-69, A70-79, A92-99, B00-09, B25-27, B33-99, D70.9, D73.3, E06.0, E32.1 |
| Acute coronary syndrome (MI) | | I20.0, I21-22, I23 |
| Other ischemic heart disease | | I20.1-9, I24-25 |
| Heart failure | | I11.0, I13.0, I13.2, I42.0-5, I42.7-9. I.43, I50, I51.7, J81, K76.1 |
| Valve disorders | | I05-I09, I34-37 |
| Other heart disease, hypertonia, cardiac surgery | | I10, I11.9, I12.9, I13.9, I15, I27.1, I27.9, I30.0, I30.8-9, I51.0-6, I51.8-9 |
| Vascular disease | | I65, I70-72, I73.1, I73.8-9, I74, I77.1, I77.6, I79.0, I79.2, K55, I87.2, I89.0, I97.2, R02, Z95.1-9 |
| Cerebrovascular disease | | G45-46, I60-64, I67, I69 |
| Thromboembolic disease | | I26, I82 |
| Arrhythmia | | I44-49 (*not included*: I46.1), R00, Z45.0, Z95.0, T82.1 |
| Pulmonary disease | | E84, I27.0, I27.2-9, I28.0, I28.8-9, J41-47, J60-J67, J68.4, J70.1, J70.3, J80, J84, J92, J95, J96, J98.2, R09.2, Z99.2 |
| Rheumatic disease | | I00-02, J99.0-1, M05-09, M12.3, M13, M30, M31.1, M31.3-9, M32-34, M35.0-3, M35.8-9, M45-46, D86 |
| Dementia | | F00-03, F05.1, G30, G31.1, G31.8-9, G32 |
| Hemiplegia, tetraplegia | | G11.4, G80-82, G83.0-3, G83.8 |
| Neurologic disease | | G10-14, G20-21, G23, G35-37, G60-62, G70-71, G91, G93.1 |
| Schizophrenia, bipolar disorder | | F20-31 |
| Other psychiatric disease | | F04, F05.0, F05.8-9. F06-07, F09, F33-39, F44.9, |
| Drug or alcohol abuse, incl. intoxication | | F10-19, K29.2, K70.0-1, G31.2, G62.1, G72.1, I42.6, R78.1-5, T40, T51, Z71.4-5 |
| Diabetes | | E10-14, I79.2 |
| Kidney disease | | N00-01, N03-07, N11-12, N17-19, N25.0, N26-27, N28.0, I12.0, I13.1, Q61.1-4, Z49, Z99.2,  *Procedure codes:* DR016, DR024 |
| Liver disease | | K70.2-4, K70.9, K71.1, K71.3-5, K71.7, K72-74, K75.2-9, K76.0, K76.2-9, R18, I81, I85, I86.4, I98.2, I98.8 |
| Gastro-intestinal disease | | K50-52 |
| Cancer, neoplasms | | C00-D48, J70.0, T45.1, Z51.0, Z51.1 (*not included*: Z85) |
| HIV/AIDS | | B20-24, F02.4, O98.7, R75, Z11.4, Z21.9, Z71.1 |
| Immune deficiency, blood disease, anaemia | | D50.1-9, D51-53, D60-61, D64.9, D65-68, D69.1, D69.3-6, D70-72, D73.0-2, D73.5-9, D76, D80-84, D89 |
| Other conditions | | G96.0, E22.2, E40-44, E46, E64, E66, E86, E87, R40.2, R64, R63.4 |
| **Medical history at any time < 5 > years**  **Main and additional discharge diagnoses**  **Inpatient and Outpatient Registry** | | |
| Any medical history | |  |
| Cardiac surgery | | *Procedure codes:* F(A-X), I97.0-1, |
| Organ transplantation | | Z94, T86, *Procedure codes:* KAS, FQA, FQB, JJC, GDG, JLE |
| Childhood conditions | | F71.1, F72-73, F79.1, F83. F84, G80, Q01-03, Q05, Q20-26, Q90 |
| **ATC-codes < 1 year**  **Drug prescription Registry** | | |
| Cardiac disease | | C09A-D, CCB, C08C, C08D, C03C, C03EB, C03A, C03B, C03D, C03EA, C07, C01B, C01AA05, C01DA, B01AC, B01AA, C10 |
| Lung disease | | R03AC, R03BB, R03BA, R03AK, R03DA |
| Diabetes | | A10B, A10A |
| Rheumatic disease | | M01A |
| Psychiatric disease | | N05A, N05B, N05C, N06A, N06D, N07BB, N07BC |
| Immune suppressive drugs | | H02AB, L01B, L04A, L01 |
| **Health care use** | | |
| No of hospitalizations in last 5 years | |  |
| No of days in hospital in last 5 years | |  |
| No of (all codes) hospitalizations in last year | |  |
| No of (all codes) outpatient visits in last year | |  |
| No of drug used in last year | |  |
| No of days of hospitalization for infectious diseases in last 5 years | |  |
| No of hospitalizations for infectious diseases in last 5 years | |  |
| No of hospitalizations in last 6 months | |  |
| **Sociodemographic factors**  **LISA registry^4^** | | |
| Age | | Age at admission |
| Sex | |  |
| County of residence | | County of residence within Sweden |
| Region of birth | | Scandinavia, rest of Europe, or rest of the world (including unknown) |
| Year of admission | | Year of sepsis admission |
| Education | | Primary, short secondary, long secondary, tertiary, or missing information |
| Occupational status | | Unemployment, employment, sickness absence, or retirement. Based on main source of income the year before sepsis. |
| Disposable income | | Disposable income the year before index date, quintiles |
| **New post-sepsis events adjusted for, from the 91-day landmark and onward** | | |
| **Medical history from day 31 to each landmark**  **Main and additional discharge diagnoses**  **Inpatient and Outpatient Registry** | **ICD-10-SE^1^, ATC^2^, or procedure^3^ code (ICD-10 codes unless otherwise specified)** | |
| Pulmonary disease | E84, I27.0, I27.2-9, I28.0, I28.8-9, J41-47, J60-J67, J68.4, J70.1, J70.3, J80, J84, J92, J95, J96, J98.2, R09.2, Z99.2 | |
| Dementia | F00-03, F05.1, G30, G31.1, G31.8-9, G32 | |
| Neurologic disease | G10-14, G20-21, G23, G35-37, G60-62, G70-71, G91, G93.1 | |
| Drug or alcohol abuse | F10-19, K29.2, K70.0-1, G31.2, G62.1, G72.1, I42.6, R78.1-5, T40, T51, Z71.4-5 | |
| Diabetes | E10-14, I79.2 | |
| Kidney disease | N00-01, N03-07, N11-12, N17-19, N25.0, N26-27, N28.0, I12.0, I13.1, Q61.1-4, Z49, Z99.2,  *Procedure codes:* DR016, DR024 | |
| Liver disease | K70.2-4, K70.9, K71.1, K71.3-5, K71.7, K72-74, K75.2-9, K76.0, K76.2-9, R18, I81, I85, I86.4, I98.2, I98.8 | |
| Cancer, neoplasms | C00-D48, J70.0, T45.1, Z51.0, Z51.1 (*not included*: Z85) | |
| HIV/AIDS | B20-24, F02.4, O98.7, R75, Z11.4, Z21.9, Z71.1 | |
| Immune deficiency, blood disease, anaemia | D50.1-9, D51-53, D60-61, D64.9, D65-68, D69.1, D69.3-6, D70-72, D73.0-2, D73.5-9, D76, D80-84, D89 | |
| Bone marrow, stem cell transplantation | DR046-DR048 | |
| Organ transplantation | Z94, T86, *Procedure codes:* KAS, FQA, FQB, JJC, GDG, JLE | |
| Childhood conditions | F71.1, F72-73, F79.1, F83. F84, G80, Q01-03, Q05, Q20-26, Q90 | |
| **ATC-codes < 1 year**  **Drug prescription Registry** |  | |
| Lung disease | R03AC, R03BB, R03BA, R03AK, R03DA | |
| Diabetes | A10B, A10A | |
| Immune suppressive drugs | H02AB, L01B, L04A, L01 | |
| Dementia | N06D | |
| Notes.  ^1^ <https://www.socialstyrelsen.se/statistik-och-data/klassifikationer-och-koder/icd-10/>, accessed 2023-10-11  ^2^ <https://www.whocc.no/atc_ddd_index/>, accessed 2023-10-11  ^3^ <https://www.socialstyrelsen.se/statistik-och-data/klassifikationer-och-koder/kva/>, accessed 2023-10-11  ^4^ Longitudinal integrated database for health insurance and labour market studies | | |

## Supplementary Methods 1. Description of cardiovascular disease risk score and definition of underlying heart disease

Outlines the cardiovascular disease (CVD) risk score methodology and provides criteria for defining underlying heart disease. Supplementary Table 3 details variables used in the model and provides additional context for stratifying sepsis patients.

### Cardiovascular disease risk score

To construct a cardiovascular disease risk score for higher risk of cardiovascular events we employed a backwards selection procedure with a cut off *p*-value of <0.15 in Cox regression models. Any cardiovascular event was used as outcome and the total follow-up time since index as time variable, and only controls were included. Variables used in the selection procedure are listed in Supplementary Table 3. The disease risk score is used to stratify cases according to risk of cardiovascular events (see results in Supplementary Figure 2)

### Underlying heart disease

Definition of no underlying heart disease included no record of *any* of the diseases or filled prescriptions listed in Supplementary Table 3. The definition is used to stratify cases according to the presence of underlying heart disease (see results in Supplementary Figure 2)

### Supplementary Table 3. Variables included in the cardiovascular disease risk score and for definition of underlying heart disease

| **Variable** | **Definition** | **Included in final model for cardiovascular disease risk score** |
| --- | --- | --- |
| Sex | Male vs female |  |
| Age | continuous | Yes |
| Myocardial infarction | Yes/no: I20.0, I21-22, I23 | Yes |
| Heart failure | Yes/no: I11.0, I13.0, I13.2, I42.0-5, 142.7-9, I43, I50, I51.7, J81, K76.1 | Yes |
| Vascular disease | Yes/no: I65, I70-72, I73.1, I73.8-9, I74, I77.1, I77.6, I79.0, I79.2, K55, I87.2, I89.0, I97.2, R02, Z95.1-9 | Yes |
| Cerebrovascular disease | Yes/no: G45-46, I60-64, I67, I69 | Yes |
| Thromboembolic disease | Yes/no: I26, I82 | Yes |
| Arrhythmia | Yes/no: I44-49 (not included: I46.1), R00, Z45.0, Z95.0, T82.1 | No |
| Number of heart medications - Filled prescription | Yes/no: C09A-D, CCB, C08C, C08D, C03C, C03EB, C03A, C03B, C03D, C03EA, C07, C01B, C01AA05, C01DA, B01AC, B01AA, C10 | Yes |
| Other ischemic heart disease | Yes/no: I20.1-9, I24-25 | No |
| Valve disorder | Yes/no: I05-09, I34-37 | No |
| Other heart disease, hypertonia, cardiac surgery | Yes/no: I10, I11.9, I12.9, I13.9, I15, I27.1, I27.9, 130.0, 130.8-9, I51.0-6, I51.8-9 | No |
| Arrhythmia | Yes/no: I44-49 (not included: I46.1), R00, Z45.0, Z95.0, T82.1 | No |
| Note. Records from Inpatient and outpatient specialists care, main and additional discharge diagnoses. Events up to ten years prior to index date included. Filled prescription drugs were included if they were prescribed up to one year prior to index date. | | |

## Supplementary Methods 2. Explorative model for identifying risk markers of CVD in sepsis patients

This appendix describes the Lasso Cox regression model used to identify potential risk markers of cardiovascular events.

1. Data preparation: The continuous variables included in the SAPS3 were categorized according to the SAPS3 scoring scale. To avoid excluding individuals with missing data on any one of the risk markers, we also used indicator variables for missing values.
2. Model development: The population was stratified into a training (80%) and a testing set (20%). We applied Lasso Cox regression on the training set to identify risk markers associated with CVD. To optimize the tuning parameter, lambda, we performed 10-fold cross-validation and selected the value that minimized estimated margin-based loss. The selected variables were then used as covariates in a Cox regression model on the testing set.
3. Outcome analysis: We first ran a Lasso Cox regression with any CVD event during days 31-365 as the outcome (n=281 events), and then separately for myocardial infarction (n=100 events), heart failure (n= 151 events), and cerebral infarction (n=43 events).

### Supplementary Table 4. Variables included in the Lasso Cox Regression

| **Baseline demographics and comorbid burden** | |
| --- | --- |
| Age | 10-year age bands |
| Sex | Female |
| Educational attainment^1^ | Primary (reference), short secondary, long secondary, or tertiary (or missing). |
| Income^1^ | Total disposable income per consumption unit, five categories based on quintiles (lowest category reference) |
| Country of birth^1^ | Sweden (reference), Nordic countries except Sweden, or non-Nordic countries |
| Occupational status^1^ | Employment (reference), unemployed, sickness absence, or retirement |
|  |  |
| **Comorbidity burden – main and additional codes in inpatient and specialist outpatient care** | |
| Myocardial infarction^2^ | Any, no/yes: I20.0, I21-22, I23 |
| Other ischemic heart disease^2^ | Any, no/yes: I20.1-9, I24-25 |
| Heart failure^2^ | Any, no/yes: I11.0, I13.0, I13.2, I42.0-5, 142.7-9, I43, I50, I51.7, J81, K76.1 |
| Valve disorder^2^ | Any, no/yes: I05-09, I34-37 |
| Other heart disease, hypertonia, cardiac surgery^2^ | Any, no/yes: I10, I11.9, I12.9, I13.9, I15, I27.1, I27.9, 130.0, 130.8-9, I51.0-6, I51.8-9 |
| Vascular disease^2^ | Any, no/yes: I65, I70-72, I73.1, I73.8-9, I74, I77.1, I77.6, I79.0, I79.2, K55, I87.2, I89.0, I97.2, R02, Z95.1-9 |
| Cerebrovascular disease^2^ | Any, no/yes: G45-46, I60-64, I67, I69 |
| Thromboembolic disease^2^ | Any, no/yes: I26, I82 |
| Arrhythmia^2^ | Any, no/yes: I44-49 (not included: I46.1), R00, Z45.0, Z95.0, T82.1 |
| Number of heart medications^3^ | Number of filled prescriptions: C09A-D, CCB, C08C, C08D, C03C, C03EB, C03A, C03B, C03D, C03EA, C07, C01B, C01AA05, C01DA, B01AC, B01AA, C10 |
| Kidney disease^2^ | Any, no/yes: N00-01, N03-07, N11-12, N17-19, N25.0, N26-27, N28.0, I12.0, I13.1, Q61.1-4, Z49, Z99.2,  *Procedure codes:* DR016, DR024 |
| Liver disease^2^ | Any, no/yes: K70.2-4, K70.9, K71.1, K71.3-5, K71.7, K72-74, K75.2-9, K76.0, K76.2-9, R18, I81, I85, I86.4, I98.2, I98.8 |
| Pulmonary disease^2^ | Any, no/yes: E84, I27.0, I27.2-9, I28.0, I28.8-9, J41-47, J60-J67, J68.4, J70.1, J70.3, J80, J84, J92, J95, J96, J98.2, R09.2, Z99.2 |
| Dementia^2^ | Any, no/yes: F00-03, F05.1, G30, G31.1, G31.8-9, G32 |
| Cancer, neoplasms^2^ | Any, no/yes: C00-D48, J70.0, T45.1, Z51.0, Z51.1 (*not included*: Z85) |
| Diabetes^2^ | Any, no/yes: E10-14 |
|  |  |
| **Disease characteristics** | |
| *SAPS3 Box 1* |  |
| Cancer therapy^4^ | no/yes |
| Chronic heart failure^4^ | no/yes |
| Blood malignancy^4^ | no/yes |
| Cirrhosis^4^ | no/yes |
| Cancer^4^ | no/yes |
| AIDS^4^ | no/yes |
| Use of therapeutic options prior to ICU^5^ | Vasoactive drugs, no/yes |
| *SAPS3 Box 2* |  |
| Reasons for admission: |  |
| Neurological | Any, no/yes: disturbance of consciousness (from coma to delirium), seizures, focal neurological deficit, Intracranial volume effect, other neurological reason |
| Cardiovascular | Any, no/yes: cardiac arrest, hypovolemia - non-hemorrhagic shock, hypovolemia - hemorrhagic shock, septic shock, cardiogenic shock, anaphylactic shock, mixed or undefined shock, chest pain, hypertensive crisis, arrhythmia, cardiovascular failure without shock, other cardiovascular reason |
| Renal | Any, no/yes: kidney failure, prerenal kidney failure, postrenal kidney failure, other renal reason |
| Respiratory | Any, no/yes: acute lung failure – Acute respiratory distress syndrome (ARDS), acute lung failure in chronic lung failure, other respiratory lung failure |
| Hepatic | Any, no/yes: liver failure, other lever reason |
| Hematological | Any, no/yes: bleeding disorder, severe hemolysis, other hematological reason |
| Metabolism | Any, no/yes: acid-base and/or electrolyte disturbance, hypo/hyperthermia, hypo/hyperglycemia, other metabolic reason |
| Gastrointestinal | Any, no/yes: gastrointestinal bleeding, acute abdomen, pancreatitis, other gastrointestinal reason |
| Trauma | No trauma, trauma |
| Other | No other, other reason |
| Acute infection at admission^1^ | Nosocomial, |
| *SAPS3 Box 3* |  |
| Level of awareness | Conscious, unconscious (Glasgow Coma Scale >13, <13; Reaction Level Scale 85: <2, >2), missing information |
| S-Bilirubin (highest), µmol/l^1^ | <34.2, 34.2 <102.6, ≥102.6, missing information |
| Body temperature, (highest) ^1^, ℃ | <35, >35, missing information |
| S-Creatinine (highest) µmol/l^1^ | <106.1, 106.1<176.8, 176.8<309.4, ≥309.4, missing information |
| Heart rate (highest) ^1^ | <120, 120<160, ≥160, missing information |
| B-leukocyte (highest), 10^9^/l^1^ | <15, >15, missing information |
| pH (lowest)^1^ | <7.25, >7.25, missing information |
| B-thrombocytes (lowest), 10^9^/l^1^ | <20, 20-49, 50-99, >100, missing information |
| Systolic blood pressure (lowest), mm Hg^1^ | <40, 40<70, 70<120, ≥120, missing information |
| Oxygenation, kPa^1^ | PaO_2_/FiO_2_<13.3 and mechanical ventilation/CPAP, PaO_2_/FiO_2_≥13.3 and mechanical ventilator CPAP, PaO_2_<8, PaO_2_≥8, missing information |
| Note. SAPS3: Simplified Acute Physiology Score 3  ^1^A dummy variable was created for each category, indicating ”Yes”  ^2^Events up to ten years prior to index date included  ^3^ Filled prescription drugs were included if they were prescribed up to one year prior to index date.  ^4^ Comorbidities ascertained by treating physician at the ICU  ^5^ Continuous infusion of adrenaline, dobutamine, dopamine (5 μg/kg/min or more), levosimendan, milrinone, noradrenaline, or vasopressin for >1 hour before intensive care  ICU = intensive care unit, PaO_2_ = partial pressure of arterial oxygen, FiO_2_ = Fraction of inspired oxygen, CPAP = continuous positive airway pressure,  In SAPS3, intrahospital location before ICU admission and time at the hospital before ICU admission are included in Box 1. However, for this analysis, they were excluded because the study's inclusion criteria specified that patients had not received treatment at the hospital before the sepsis event. In Box 2, the type of surgery is normally included but was excluded from this analysis as 99.7% of cases did not have surgery. | |

# Supplementary Results

## Supplementary Table 5. Details of risk time and number of cardiovascular events and observations

*
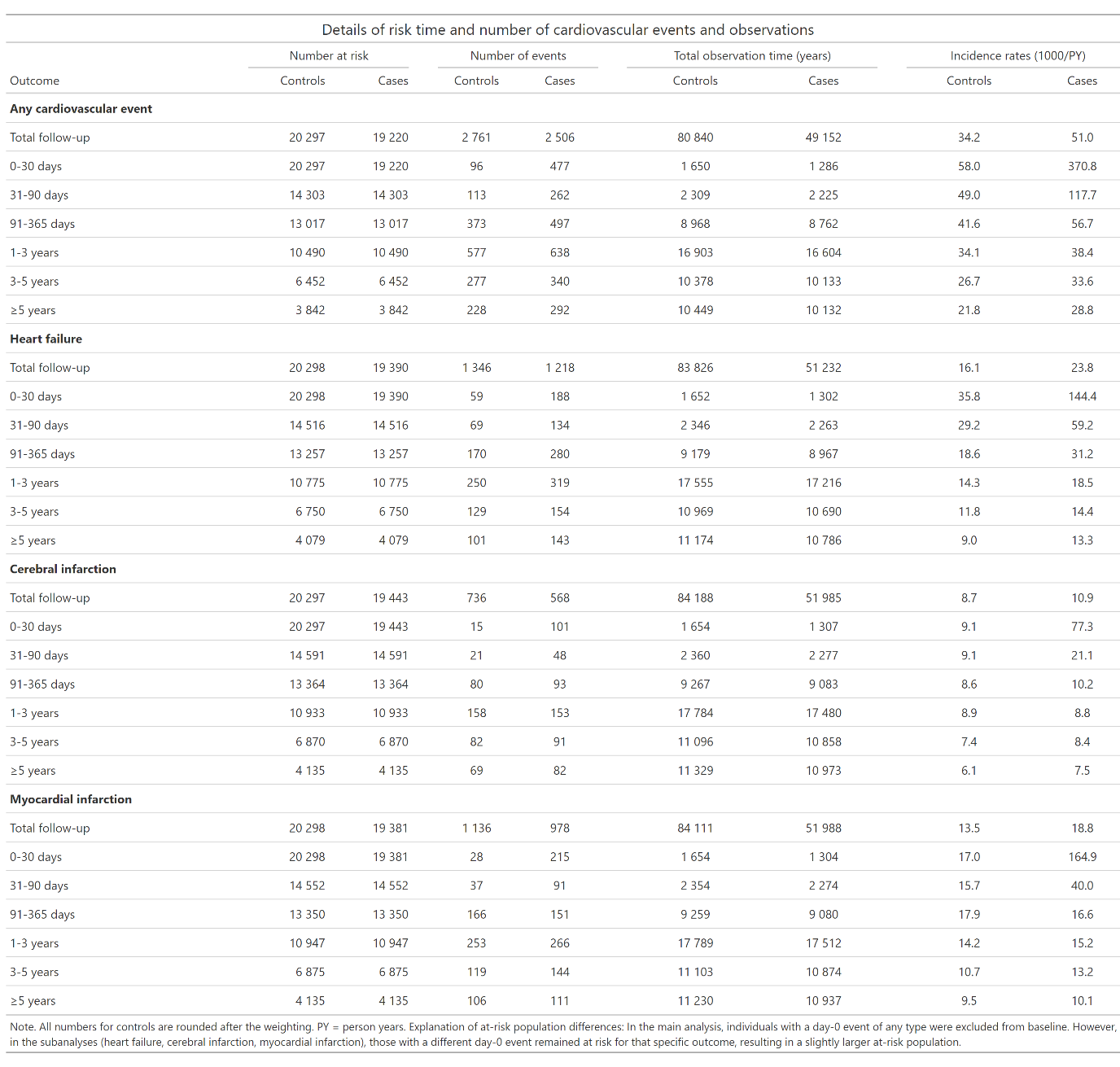
*

Supplementary Table 6. Absolute risks and risk differences for CVD


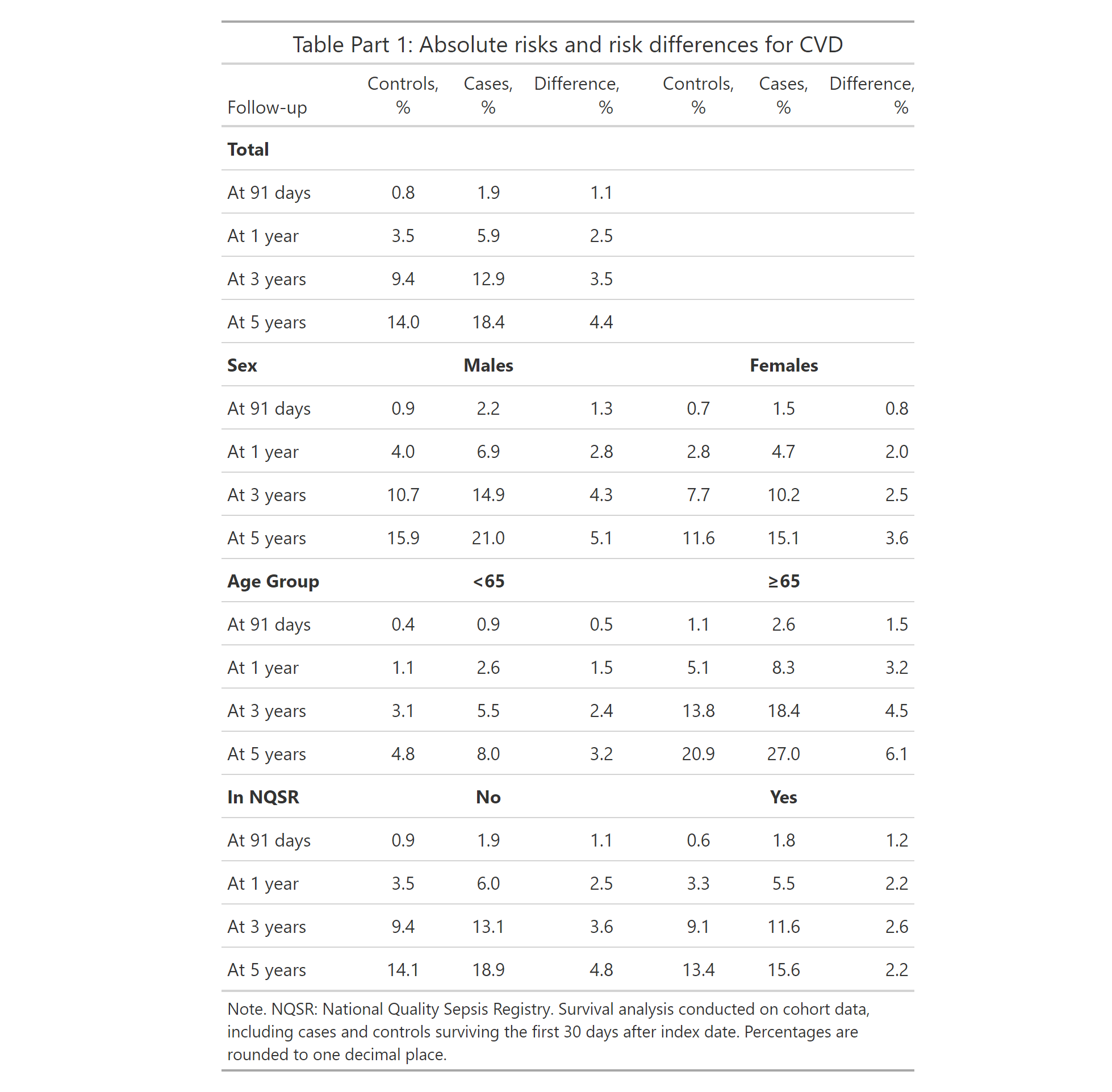


#


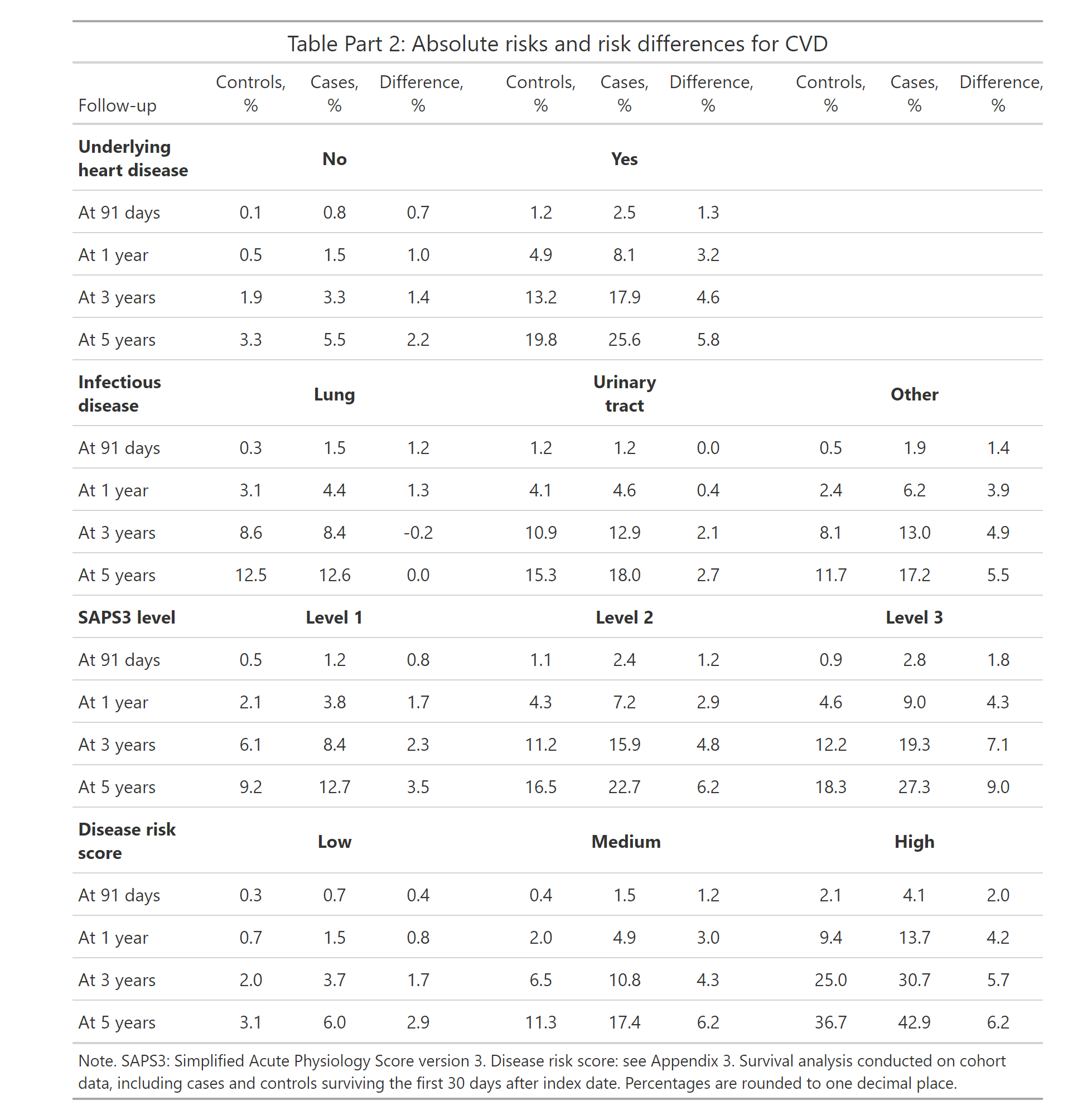


## Supplementary Figure 1. Sensitivity analysis: Comparison of hazard ratios for cardiovascular events using different weighting methods


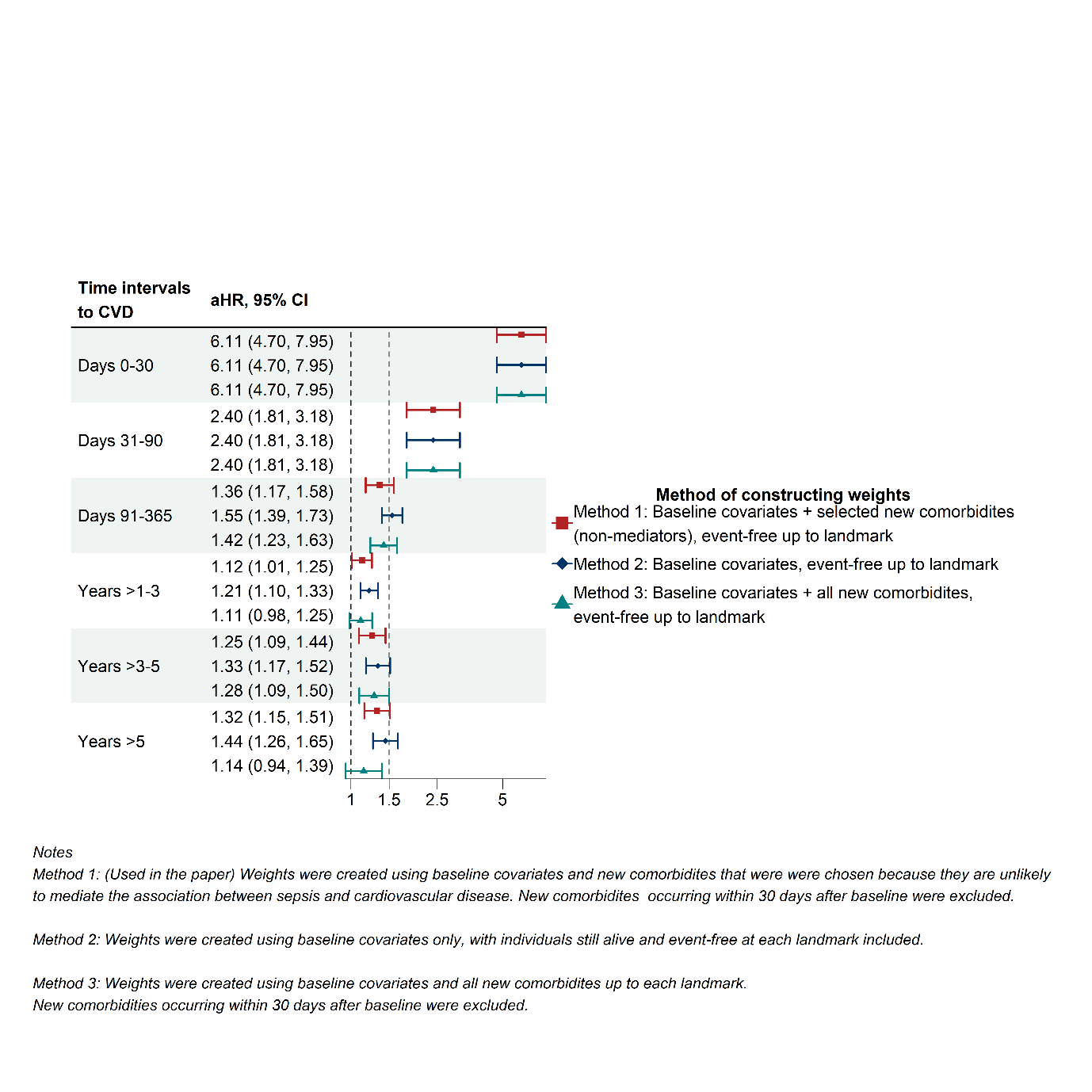


## Supplementary Table 7. Test of interaction between sepsis status and calendar year

Across all landmark intervals (0–30 days, 31–90 days, 91–365 days, 1–3 years, 3–5 years, and >5 years), no statistically significant interaction between sepsis status and calendar year was observed (all global Wald tests p > 0.05), indicating that the relative risk estimates were stable over calendar time.

Global Wald test: interaction sepsis × admission year

---------------------------------------------------

df Wald chi2 p-value

---------------------------------------------------

0–30d 11 12.46 .330

31–90d 11 9.69 .558

91–365d 11 14.73 .195

1–3y 10 11.12 .348

3–5y 8 6.46 .596

>5y 6 12.43 .053

---------------------------------------------------

## Supplementary Figure 2. Weighted hazard ratios for subgroup analyses


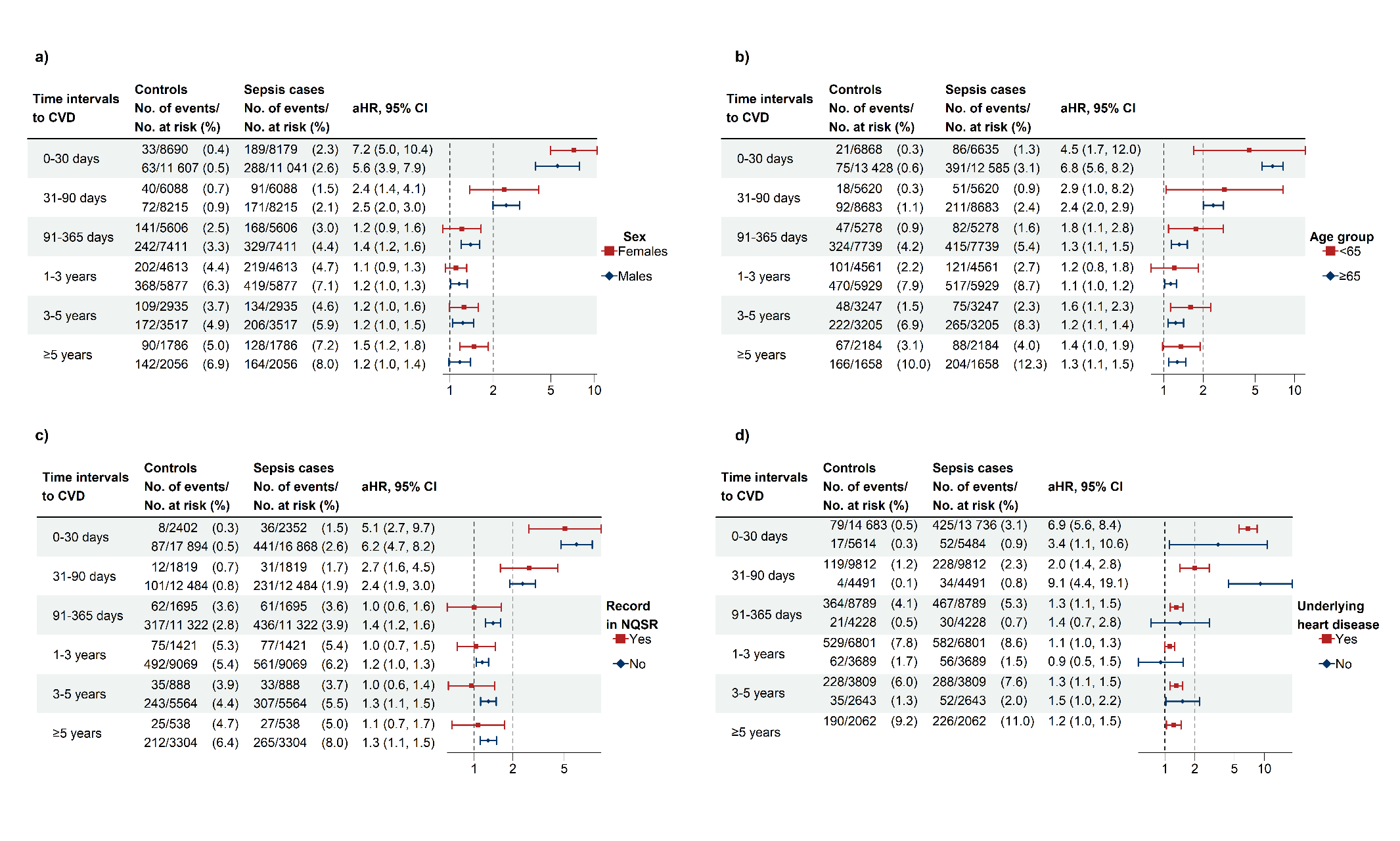


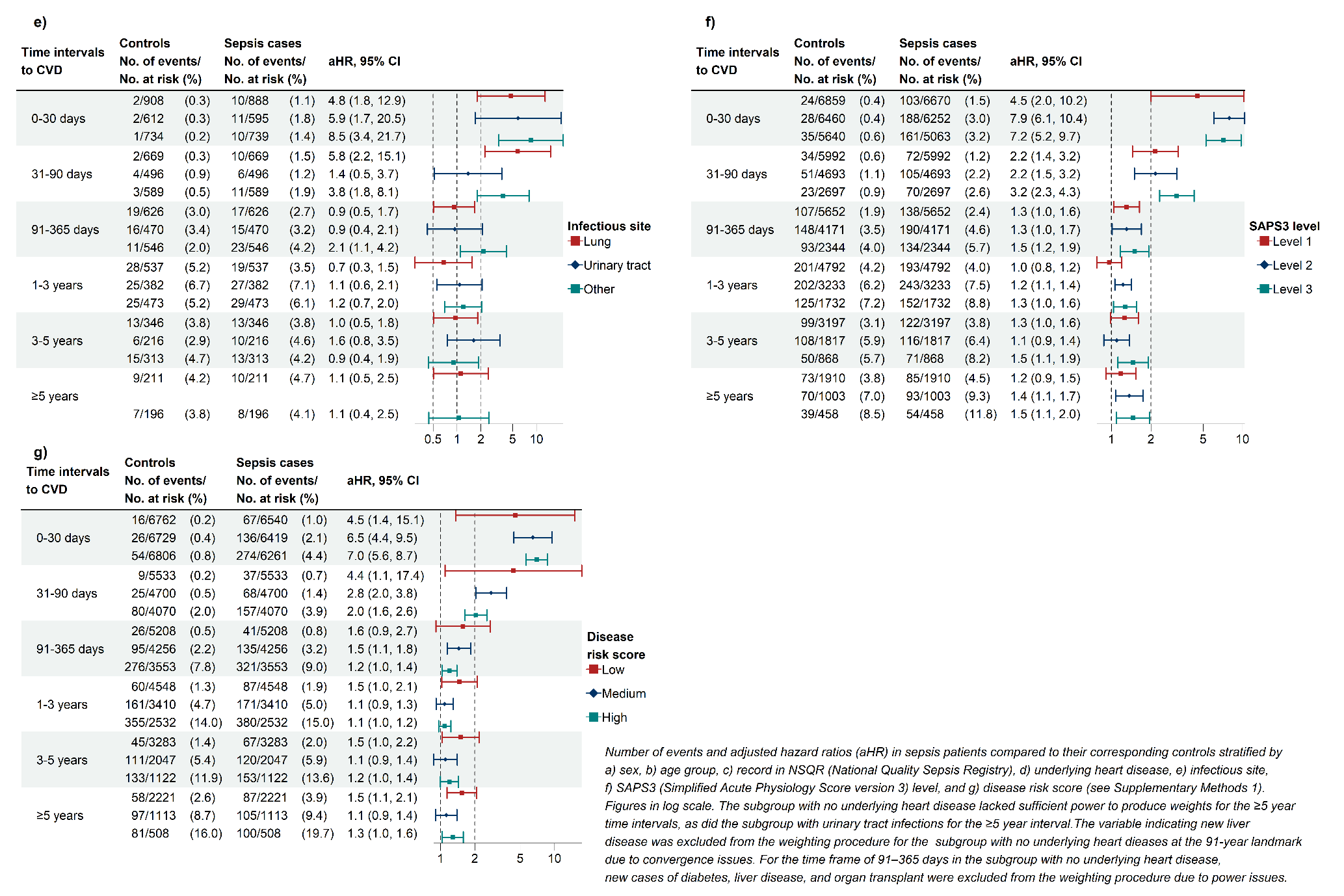


##
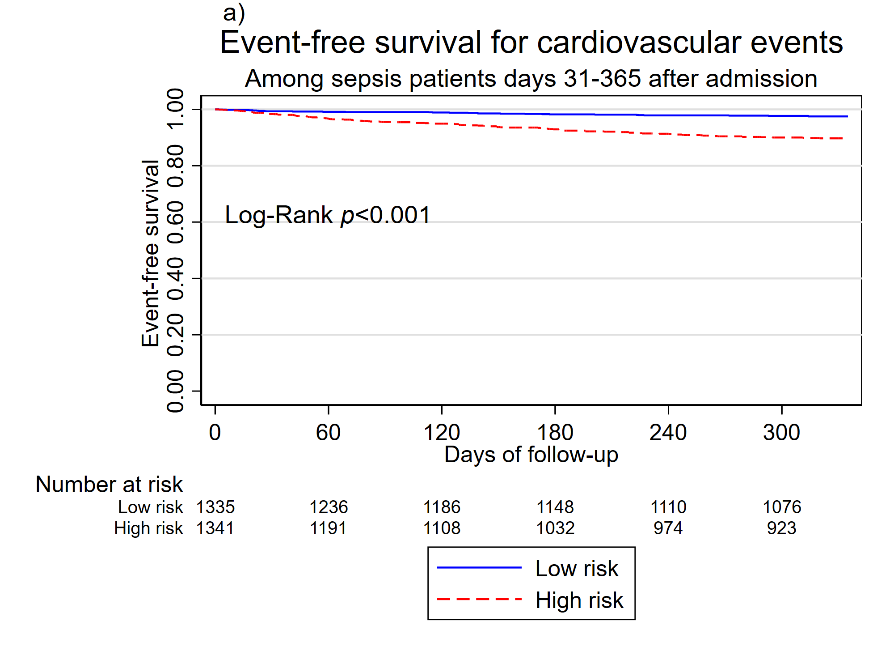

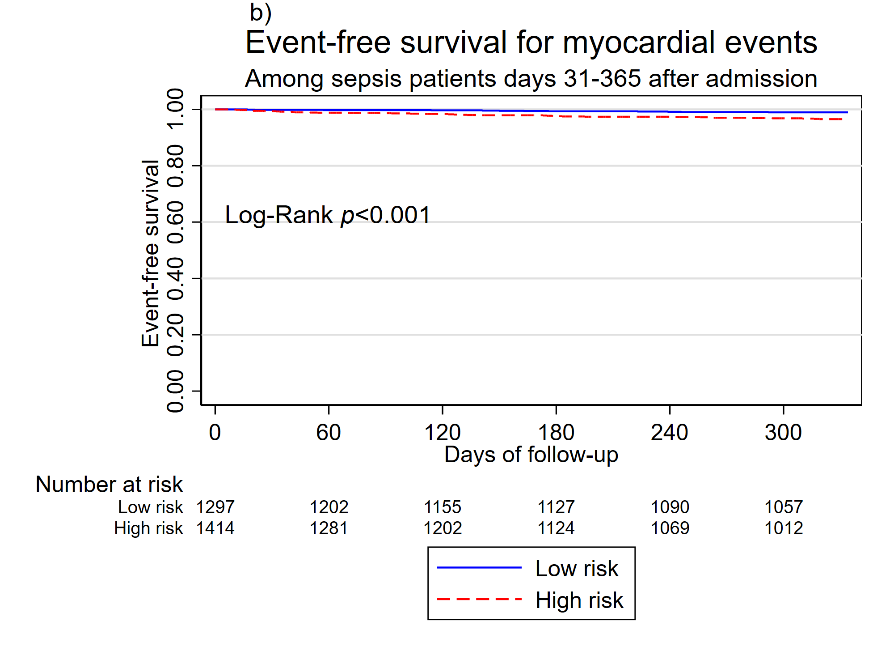

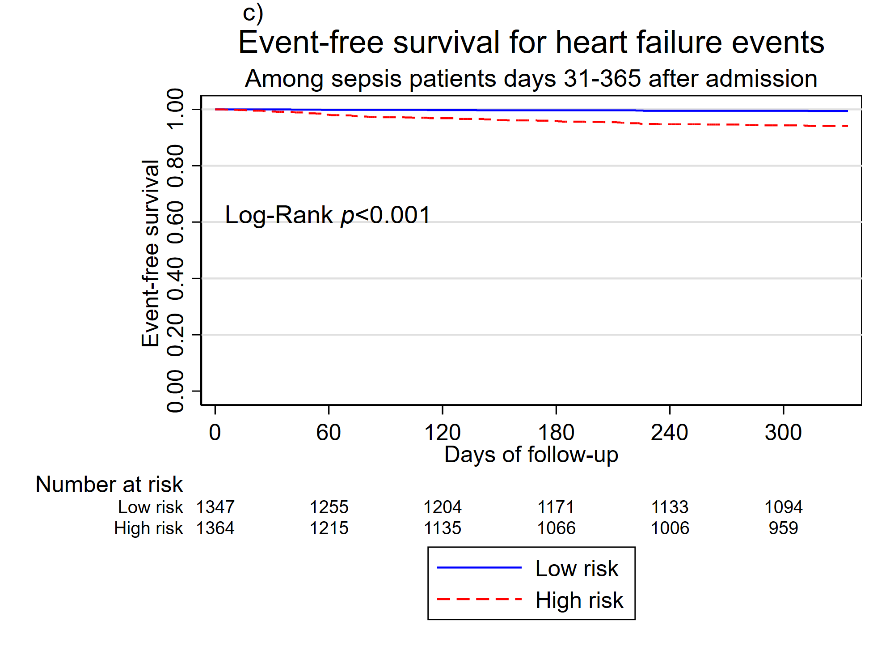

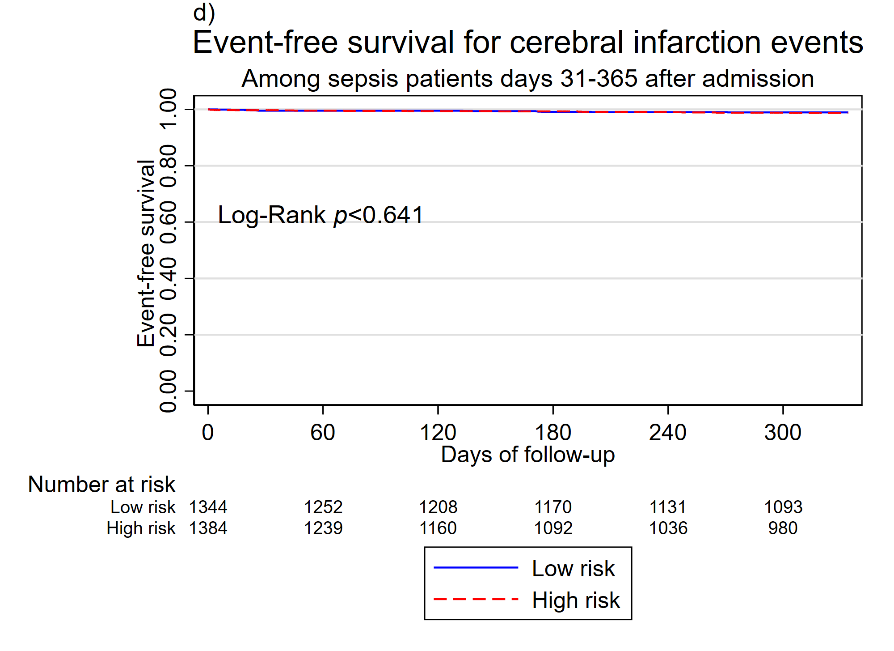
Supplementary Figure 3. The prognostic capacity of the models selected by Lasso regressions

*Supplementary Figure 5. The testing set was stratified into low- and high-risk groups based on the median risk score derived in the Lasso Cox regression in the training set. The Kaplan–Meier survival analyses confirmed the high-risk group yielding reduced survival time (p<0.001) for the composite outcome cardiovascular events, myocardial infarction, and heart failures, but not for cerebral infarctions (p=0.641): a) cumulative cardiovascular events, b) cumulative myocardial events, c) cumulative heart failure events, and d) cumulative cerebral infarctions.*

## Risk markers by type of event, as selected by Lasso Cox regression

### Supplementary Figure 4. Risk markers for any cardiovascular event 31-365 days after admission


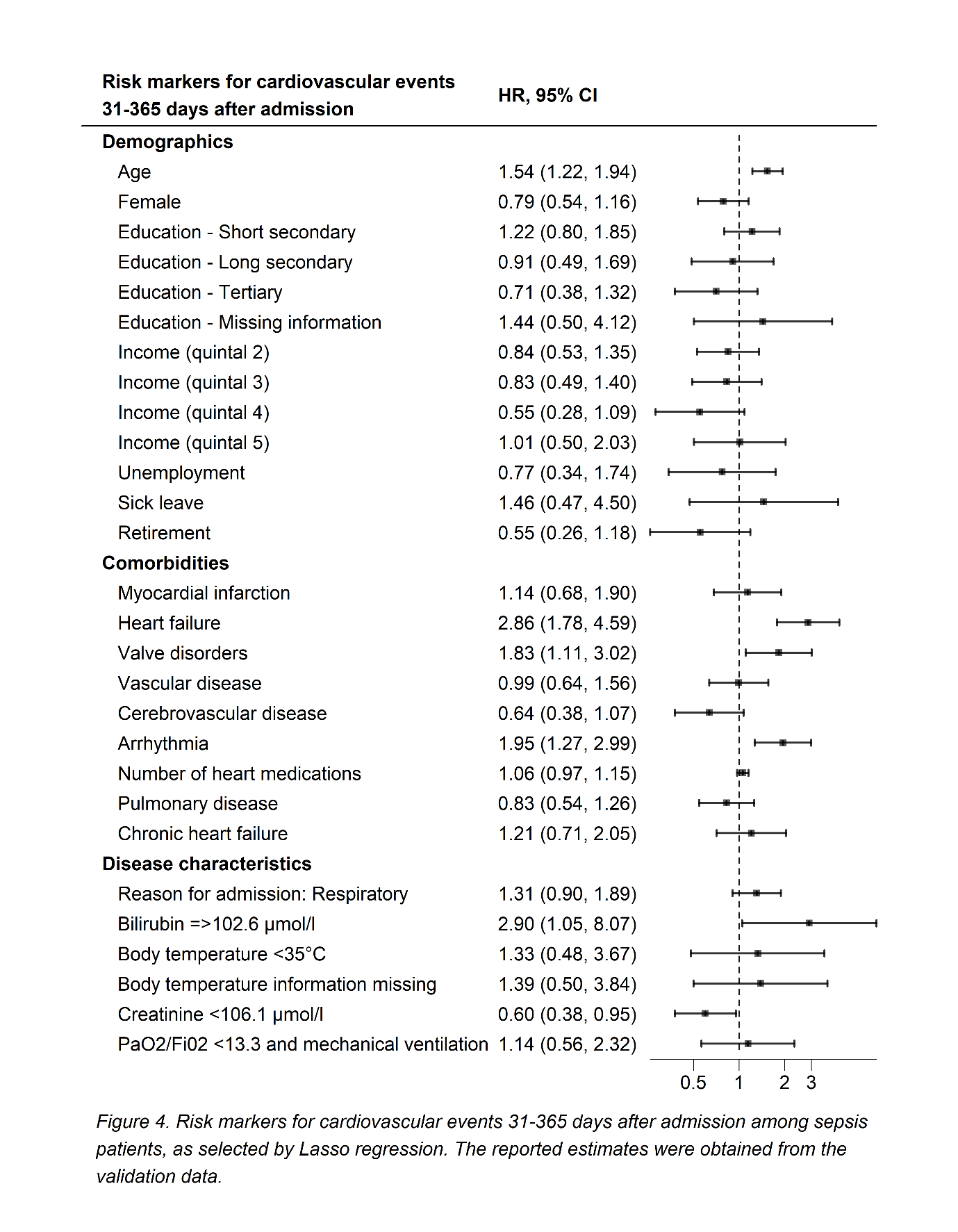


### Supplementary Figure 5. Risk markers for myocardial infarction 31-365 days after admission


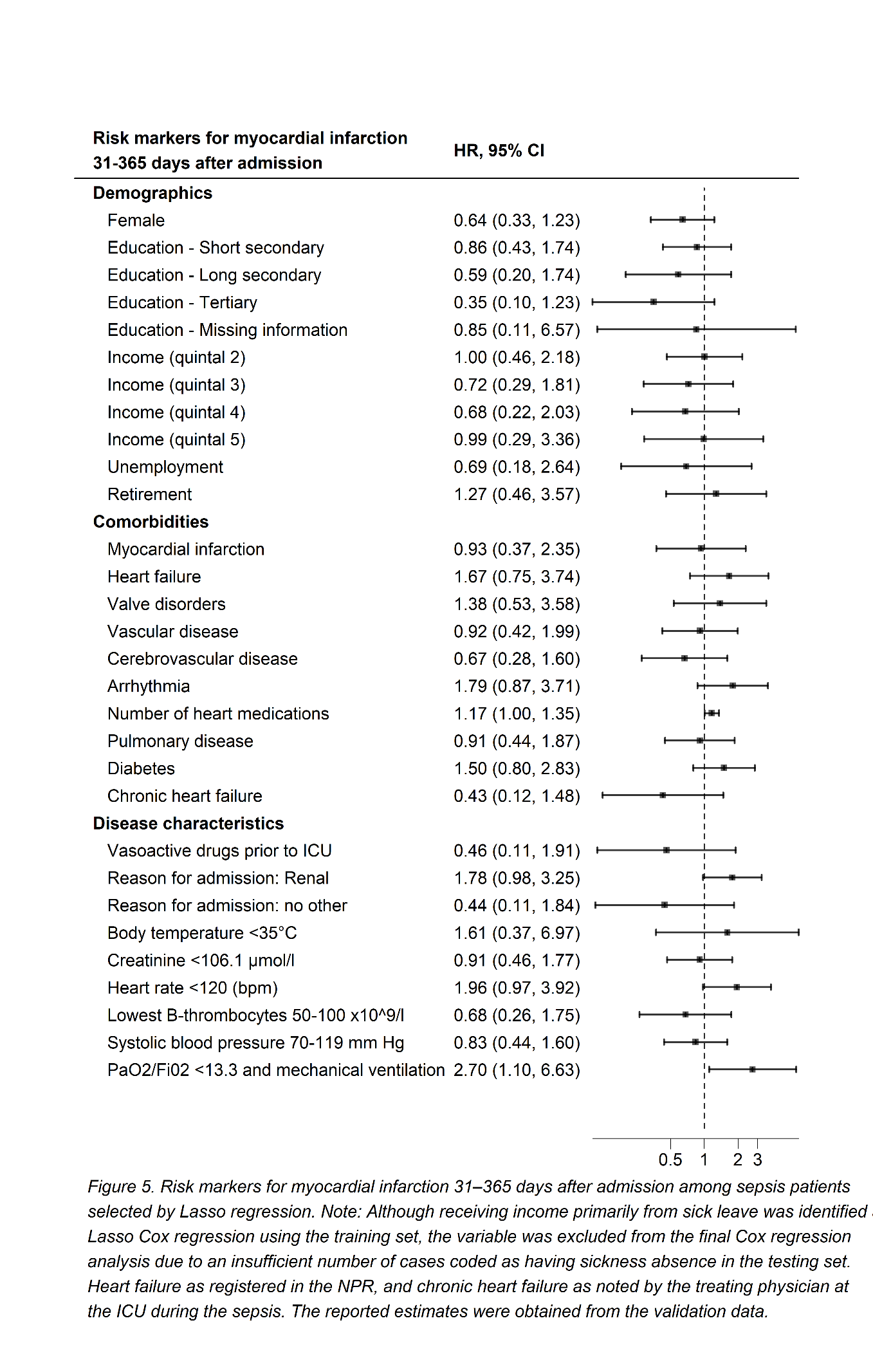


### Supplementary Figure 6. Risk markers for heart failure 31-365 days after admission


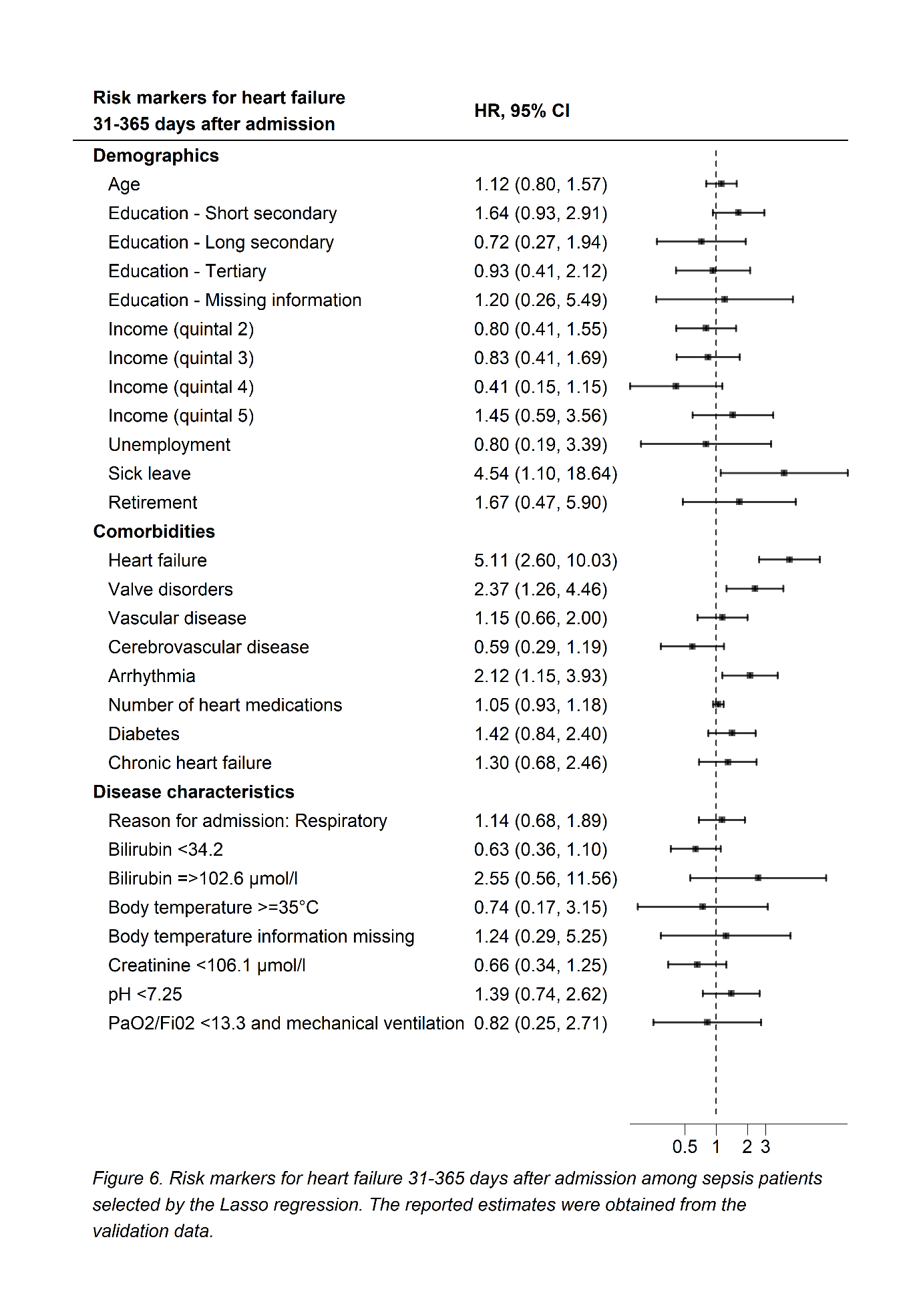

Supplement: Wetterberg et al. supplementary material [file S0950268826101174sup001.docx]
